# Twenty-first Announcement of the Medical Dept. Iowa State University

**Published:** 1867-12

**Authors:** 


### IOWA MEDICAL JOURNAL.

VOL. V. *November & December*, 1867. No. 1.

TWENTY-FIRST ANNOUNCEMENT

OF THE

### Medical Department of the Iowa State University.

**Located at the City of Keokuk, Iowa.**

---

*President of University.*—Rev. O. M. SPENCER, D.D.

*Curators of Medical Department.*—E. R. FORD, M.D., Prest.; E. H. HARRISON, Sec'y; WM. LEIGHTON, Esq., Hon. SAMUEL F. MILLER, WM. PATTERSON, Esq., Hon. R. P. LOWE, S. HAMILL, Esq., J. B. HOWELL, Esq.

*Medical Faculty.*—

J. C. HUGHES, M. D.,
**Professor of the Institutes and Practice of Surgery and of Surgical Clinics.**

GEORGE W. HALL, M. D.,
**Professor of Physiology, Pathology and General Therapeutics.**

H. T. CLEAVER, M. D.,
**Professor of Obstetrics and Diseases of Women and Children.**

A. M. CARPENTER, M. D.,
**Professor of the Institutes and Practice of Medicine and of Medical Clinics.**

E. J. GILLETT, M. D., D. D.,
**Professor of Chemistry, Toxicology, Materia Medica and Microscopy.**

EDWARD CLAPHAM, M. D.,
**Professor of General and Microscopal Anatomy, and Demonstrator.**

---

D. MOOAR, LL.D.,
**Lecturer on Medical Jurisprudence and Forensic Toxicology.**

L. C. INGERSOLL, M. D.,
**Lecturer on the Principles of Dental Science.**

*Dean of the Faculty.*—J. C. HUGHES, M. D.

### ANNOUNCEMENT.

The Session of 1867–8 will commence on Wednesday, October 30th.

The Iowa State University was created by Act of Legislature during the Session of 1846–47, and was munificently endowed by an appropriation from the General Government. This, the Medical Department, was established by Act of Legislature in the year 1849, and has been liberally assisted by appropriations from the State.

The Faculty of the Medical Department of the Iowa State University are pleased to announce to the profession throughout the Northwest, that the *Twenty-first* regular course of lectures of an Institution whose triumph has been signal, will open on Wednesday evening, October 30th, with a general introductory lecture, by Prof. Gillett. The session will close on the last day of February.

The College Clinic affords ample opportunities for the student to apply the principles which he derives from the various branches taught. Patients are examined almost daily in the presence of the Class, and surgical operations performed (if required) or prescribed for; and here the Professor conducting the clinic elaborates in detail, and explains the *modus operandi* of prescriptions, and the process of cure wrought by appliances and surgical operations. The surgical clinic is conducted twice a week, and frequently daily, by the Professor of Surgery, while the medical clinic is provided for by the Professor of Practice.

By reference to the resolutions passed by the Teachers' Convention, held at Cincinnati during the session of the American Medical Association in May last, it will be observed that the length of time recommended for study is increased to four years—including three lecture terms consisting of six months each—before the student is admitted to an examination for the degree of Doctor of Medicine. While the Faculty most cordially indorse the suggestions of the Teachers' Convention with a view to the elevation of the standard of medical education, we deem it best to adhere, for the present Session, to the established usage of colleges throughout the country, which require three years study, including two courses of medical lectures—or, as an equivalent, four years reputable practice and one course of lectures—as a pre-requisite.

Students are requested to make their arrangements to be present at the opening and remain until the close of the session; and the better to secure this end, the Faculty would here state that certificates of attendance will be issued *only* for the time actually spent in attendance upon lectures.

FEES.

| | |
|---|---|
| For the entire Course of Instruction, - - | $40 00 |
| Matriculation Ticket, - - - - - - - - | 5 00 |
| Demonstrator's Ticket, - - - - - - - | 5 00 |
| Hospital Tickets, *gratuitous.* | |
| Graduation fee, - - - - - - - - - | 30 00 |

The total expense to the student is less than at any other School of the country.

Graduates of this, and other regular schools of medicine, are admitted to all lectures, upon payment of a matriculation fee of ten dollars.

REQUIREMENTS FOR GRADUATION.

Each student is required, within one week after the opening of the session, to pay the fees and procure his matriculation ticket. Candidates for graduation—

1st. Must be twenty-one years of age, and present testimonials of good moral character.

2d. Must have attended two full courses of medical lectures, the last at the Medical Department of the Iowa State University; or, evidence of four years' reputable practice will be considered as equivalent to one course.

3d. Must have studied medicine three years (including lecture terms) under the direction of a respectable medical practitioner.

4th. Must furnish a satisfactory medical thesis (original and in his own hand-writing), to be delivered to the Dean, at least four weeks before the close of the session, accompanied by the graduation fee.

5th. Must pass a satisfactory examination by the Faculty, at the close of the session.

The attention of students is called to the fact that the session of four months—six lectures daily—equals, in amount of instruction, any other school in the country.

The Hospitals located in this city give superior clinical advantages to the student, and the moderate cost of tuition and other expenses, make it one of the most desirable points for a thorough medical education.

### TEXT BOOKS.

Students would do well to furnish themselves with *some* of the following works on the different departments:

*Anatomy*—Wilson, Cruveilhier, Gray.
*Chemistry*—Graham, Turner, Lehmann.
*Physiology*—Draper, Carpenter, Dalton.
*Practice*—Wood, Watson, Flint, Bennett.
*Materia Medica*—Wood's Therapeutics, U. S. Dispensatory.
*Surgery*—Miller, Chelius, Erichsen, Druitt, Pirrie or Gross.
*Pathological Anatomy*—Gross, Rokitansky, Gluge.
*Diseases of Women and Children*—Churchill, Hodge.
*Obstetrics*—Cazeau, Meigs, Ramsbotham, Churchill, Hodge.
*Practical Anatomy*—Agnew, Dublin Dissector, Wilson's Dissector.
*Medical Jurisprudence*—Dean, Taylor, Beck.
*Microscopy*—Peaslee's Histology, Hassall.
*Pathology*—Copland, Hurle, Simon, Williams.

Board can be obtained in the city at from three to four dollars per week. Students, upon their arrival, can obtain information as to good boarding houses, by calling at the office of the Dean, on Second street, near the Deming House, or the Janitor who resides in the College.

Medical books may be purchased in our book stores on as good terms as in any eastern city.

### NEW BUILDING.

Our college edifice, erected during the summer of 1858, and dedicated by his Excellency, Governor Lowe, to the object and purposes of medical teaching, has done much to increase the permanency and prosperity of this department of our University.

The building is admirably adapted to the purposes of medical teaching, being in every way equal to the wants of the profession and student. The accompanying cut gives an accurate idea of its exterior; its interior arrangement is not surpassed by any institution east or west. It occupies a central position in the city, and presents a front of 90 feet by 65 in depth, and is four stories high. Upon the first floor a City Dispensary, where the poor receive gratuitous advice and attention from the different members of the Faculty, offering to the student superior advantages for clinical instruction. Upon the second floor, a large Lecture Room, Chemical Laboratory Apparatus Room; on the opposite side of the hall, a commodious Museum, already furnished with rare and valuable specimens, large additions having been made by Professor Hughes, which furnishes to the student means of demonstration not surpassed by any institution of the country. There are also two fine rooms in front, occupied as Faculty and Library Rooms. In addition to these, two rooms for the study of practical Anatomy. On the next floor, an Amphitheatre for anatomical and surgical demonstrations; this room is 40 feet in its traverse diameter, with a vertical diameter of 42 feet, extending through both stories and lighted from the dome, and so arranged that every student may witness the most delicate operation.

### APPLIANCES FOR TEACHING.

The various departments are fully supplied with the means necessary for illustrative teaching. The Chair of Materia Medica is furnished with a full suit of *pure* and *adulterated* drugs, giving to the student ample means of qualification in his selection of unadulterated medicines. Also one of the largest collections of *Floral* and *Medical Botany* to be found in any school of the country.

Physiology, Pathology, and Practice are amply supplied. Specimens of disease, both wet and dry, have been largely increased, and every variety of skin disease fully illustrated. The Chair of Anatomy presents not only the *cadaver*, but demonstrates, from models of the most improved and delicate workmanship (both of French and American artists), the eye, the ear, the brain, nerves, arteries and veins, and, indeed, every part of the organism. The Chair of Obstetrics is fully represented by manikins and models, giving to the student every advantage obtained from teachings in this branch of science.

The Faculty are in receipt of a large and handsome collection of Pathological specimens, illustrative of almost every morbid condition of the visceral organs, as a donation to the Museum from their colleague, Prof. M. K. Taylor, which, together with the extensive collection of Anatomical, Philosophical and Pathological specimens purchased by Prof. Hughes during his sojourn in Europe, affords to the student extraordinary facilities for the perfection of his art.

The department of the principles of Dental Science, is a new feature in our school since the last session. This has been brought about by concert of action between the Faculty and the Iowa State Dental Society. This department will embrace the following subjects, viz.: Anatomy, Histology, Physiology, Pathology, Hygiene and Therapeutics, so far as they relate to the teeth, and will be taught independent of the regular course. The fee for this course will be $5 00, and is optional with the student.

### CLINICAL MEDICINE AND SURGERY.

The hospitals of the city offer superior advantages to the student in the study of Clinical Medicine and Surgery. The City Hospital and Dispensary, our County Alms House, in the vicinity of the city, and our College Infirmary, furnish abundant means for observation and the study of every variety of disease. The practical members of our profession well understand that looking through the wards of large hospitals, does not add materially to the student's knowledge of disease; but it is the close study and observation in single cases that makes the true medical man.

### HUGHES' MEDICAL AND SURGICAL INFIRMARY.

This institution, connected with the College, is under the charge of the Professor of Surgery, who has refitted and newly furnished this Infirmary, and is now prepared to comfortably accommodate all patients who may present themselves, either for medical or surgical treatment.

### EYE AND EAR.

In connection with the Infirmary, a special ward for the treatment of diseases of these organs has been established, where those applying can have all the advantages, either from operation or treatment, that science affords.

The use of the *Opthalmoscope*, in diagnosing diseases of the eye, will be fully taught and demonstrated. The *Auriscope*, which is so necessary in examinations of the ear, will be particularly illustrated; and the *Laryngoscope*, which has done so much in revealing the hidden diseases of the throat and vocal cords, will receive that share of attention in the treatment of diseases of these organs, that it practically merits.

Good bath rooms are affixed, and patients presenting themselves, will have the benefit of all scientific improvements in hospital treatment.

Charges to patients will at all times be as moderate as in other institutions of a similar character. *Board and Medicine must be paid for in advance.*

During the College session of four months, commencing October 30th, all patents coming before the class will be operated upon gratuitously, *a small fee only being charged for the necessary dressings and attention;* and SOLDIERS suffering from wounds received in the service, requiring operation, and who have been honorably discharged, *will be operated upon without charge.*

### PRACTICAL ANATOMY.

The Dissecting Room is well lighted with gas, material abundant, and, in addition to the usual demonstrations, the Professor of Anatomy gives especial attention to this department by private lectures and examinations. And in order that students may fully appreciate the importance of a thorough knowledge of anatomy, relative to medicine as well as surgery, the Professor of Surgery proposes to give a prize, consisting of anatomical models to the value of $50, which shall be presented to the student who shall prepare, during the session, the best anatomical preparation, provided not less than six shall compete for it, and the specimens presented shall belong to the Museum, and there bear the name of the author.

### PRIVATE INSTRUCTION.

Courses on special diseases or subjects will be given privately by the members of the Faculty, to meet the demands of practitioners and students who may desire minute practical instruction upon branches of the science in detail.

### CATALOGUE OF STUDENTS.—SESSION 1866–7.

| | |
|---|---|
| Allen, H. W., Colorado, | Practitioner. |
| Armstrong, J. B., Iowa—Dr. J. M. Sturdevant, | Preceptor. |
| Arnold, W. M., Iowa—Dr. Atkinson, | " |
| Blakely, W. T., Iowa—Dr. Hay, | " |
| Brown, W. H., Mo.—Drs. Christy & Stutsman, | " |
| Burch, B. F., Mo.—Dr. John Cutler, | " |
| Boyer, J. M., Iowa—Dr. T. W. Boyer, | " |
| Black, W. H., Mo., | Practitioner. |
| Burgess, J., Iowa, | Iowa University. |
| Burgess, C., Iowa, | " |
| Cross, J. L., Iowa—Dr. T. W. Taylor, | Preceptor. |
| Cozad, E. F., Iowa—Dr. Hay, | " |
| Cook, J. D., Mo.—Dr. J. J. Oakes, | " |
| Clark, E., Iowa—Dr. M. W Newsome, | " |
| Crawford, S. B., Mo.—Dr. Barnett, | " |
| Cone, W. D., Iowa—Professor Cleaver, | " |
| Craven, J. T., Mo.—Dr. Henry, | " |
| Cross, H. H., Iowa, | Iowa University. |
| Campbell, A., Canada West, | " |
| Davis, A. C., Mo., | Practitioner. |
| Davis, P., M. D., Mo., | " |
| Doig, J. R., Iowa—Dr. Cowden, | Preceptor. |
| Davison, T. A., Ills.—Dr. Corkins, | " |
| Davison, W., M. D., Iowa, | Practitioner. |
| Everman, T. H., Mo.—Dr. McKim, | " |
| Fischer, F. Z., Switzerland, | Iowa University. |

| | |
|---|---|
| Forsee, B. W., Mo.—Dr. Briscow, | Preceptor. |
| Fowler, J. T., Mo.—Dr. Frazer, | " |
| Fowler, S. C., Iowa—Dr. Huster, | " |
| Glasgow. J. M., Iowa—Dr. Shrader, | " |
| Gilmer, J., Iowa—Dr. Belcher, | " |
| Harris, W. T., Ills.—Dr. Harris, | " |
| Hoxsey, A. P., Ills.—Dr. Catherwood, | " |
| Hitt, J. E., Iowa—Dr. Craig, | " |
| Horn, J. A., Mo.—Dr. Ball, | " |
| Hall, R. N., Iowa—Dr. Leslie, | " |
| Hayden, H. R., Iowa—Dr. Hayden, | " |
| Hughes, J. C., Jr., Iowa—Prof. Hughes, | " |
| Henry, Jas., Iowa—Dr. S. Henry, | " |
| Heizer, R., Iowa, | Iowa University. |
| Hill, B., Iowa—Dr. Cables, | Preceptor. |
| Huff, C. H., Mo.—Dr. Wagner, | " |
| Hopkins, W. R., Mo.—Dr. Barnett, | " |
| Hickman, J. G., Mo.—Dr. Goodyear, | " |
| Hall, W. L., " —Dr. Cash, | " |
| Jones, T. B., Ind.—Dr. Butcher, | " |
| Jones, W. M., Ind.—Dr. Butcher, | " |
| Kimmel, E. M., Mo.—Dr. Oakes, | " |
| Kirkland, J. J., " —Dr. Finley, | " |
| Sikes, F. W., Ills.—Dr. Miller, | " |
| Lionhardt, C., Iowa, | Iowa University. |
| Larimer, J. M. Ills.—Dr. Calton, | Preceptor. |
| McCutchan, J. F., Ills.—Dr. Webster, | " |
| McCassen, J. F., Ind.—Dr. McCassen, | " |
| Mead, R. H., Ky —Dr. O. J. Mead, | " |
| McDonald, J. A., Minn., | Practitioner. |
| McKee, L. D., Mo.—Dr. Tinsman, | Preceptor. |
| McCutcheon, R., West Va.—Dr. Winslow, | " |
| Morgan, Jno., Iowa—Dr. Morgan, | " |
| Morgan, J. W., Ills.—Dr. Scott, | " |
| Moor, Wm., Iowa, | Iowa University. |
| McConnaughey, D. S., Iowa, | " |
| McDannell, O. C,, Wisconsin, | Practitioner. |
| McCleary, J. D., Iowa, | " |
| Norris, S. C., Iowa—Dr. J. N. Norris, | Preceptor. |
| Newman, M. N., Iowa—Dr. J. N. Norris, | " |
| Newsome, W., Iowa—Dr. M. W. Newsome, | " |
| Norris, Jeff. T., La.—Dr. J. L. Moor, | " |
| Orr, R. O. B., Mo —Dr. Collgrove, | " |
| Overholt, D. W., Iowa, | Practitioner |
| Olive, R., Wisconsin, | " |

2

| | | |
|---|---|---|
| Oakes, J. J. Missouri., | | Practitioner. |
| Proctor, J. W., Mo., | | " |
| Peregrew, J. S., Indiana—Dr. Butcher, | | Preceptor. |
| Pollock, T. D., Iowa—Dr. O. H. Priser, | | " |
| Perkins, F. W., Mo.—Dr. Settle, | | " |
| Rogers, M., Mo., | | Iowa University. |
| Richards, W. J., Ills., | | Practitioner. |
| Sala, O. P., Iowa—Dr. Sala, | | Preceptor. |
| Stewart, F. C., Iowa—Dr. Stansberry, | | " |
| Sturgeon, W. P., Ills.—Dr. Lyman, | | " |
| Stanton, D. A., Iowa—Dr. Sturdevant, | | " |
| Stevenson, R., Iowa—Dr. Stevenson, | | " |
| Stevens, J. A., Iowa—Prof. Hughes, | | " |
| Searle, B. W., Iowa—Dr. D. A. Laforce | | " |
| Sanders, S. F., Ills.—Dr. W. T. Wright, | | " |
| Scott, M. B., Mo.—Dr. Fortner, | | " |
| Stutsman, D. W., Iowa—Dr. Stutsman, | | " |
| Stutsman, M. D., Iowa, | | Practitioner. |
| Thomas, F. S., Ills.—Dr. Hollowbush, | | Preceptor. |
| Tucker, J. M. Missouri—Dr. M. F. Tucker, | | " |
| Thompson, W. L., " —Prof. Carpenter, | | " |
| Trask, G. H., Ills.—Dr. E. H. Trask, | | " |
| Thompson, J., Iowa, " " | | " |
| Vawter, W. F., Mo.—Dr. W. R. Rodes, | | " |
| Weisman, A., M. D., Iowa. | | Practitioner. |
| Wilson, J. G., Iowa—Dr. W. H. Martin, | | Preceptor. |
| Wheeler, Charles, Iowa—Prof. Cleaver, | | " |
| Wamsley, L. B., Mo.—Dr. J. W. Lee, | | " |
| Wilson, H. H., Ills.—Dr. B. C. Toley, | | " |
| Wilhite, W. J., Mo.—Dr. Alexander, | | " |
| Wall, T. M., Iowa—Dr. John Irwin, | | " |

Medical men who are permanently located, and desire to receive the Circular regularly, are respectfully requested to forward their addresses to the Dean.

Members of the profession having medical or surgical cases in their practice, which they do not wish to treat themselves, will confer a favor upon the Faculty, and the Institution, by recommending them to our Clinics, where they can receive gratuitous advice, or operation, if required.

We would call the attention of Practitioners of Medicine, who are under-graduates, or who have attended but one course of lectures, to the fact, that the course suggested by the Convention of Colleges, and ratified by the American Medical Association, at its last session in Cincinnati, and which will, in all probability, be adopted before the session of 1868–9, makes four years' study and three courses of lectures necessary to graduation.

Those wishing to avoid the embarrassment and additional expense which this will incur, will save time and money by completing their studies as early as possible.

The following named gentlemen were admitted to the degree of *Doctor of Medicine* at the close of the last session :

GRADUATING CLASS OF 1866–7.

| NAMES OF GRADUATES. | RESIDENCE. | SUBJECT OF THESIS. |
|---|---|---|
| Allen, H. W | Valmount, Colorado | Puerperal Eclampsia. |
| Arnold, W. M | Fort Madison, Iowa | Diptheria. |
| Black, W. H | Breckinridge, Mo | Pernicious Fever |
| Buren, W. F | Trenton, Mo | Hemostatics. |
| Cone, W. D | Keokuk, Iowa | Hygrology. |
| Craven, J. T | Fairmount, Mo | Scarlatina. |
| Crawford, B. S | Greensburg, Mo | The Circulation. |
| Davis, A. C | Kirksville, Mo | Inflammation. |
| Forsee, B. W | Lima, Ills | Chronic Indigestion |
| Gillmore, J. E | Monmouth, Iowa | Eczema. |
| Larimore, J M | Warsaw, Ills | Pneumonia. |
| Mead, R. H | Huntsville, Ills | Typhoid Fever. |
| McConnaughey, D. S | Washington, Iowa | Dysmenorrhea. |
| McDonald, John S | St. Paul, Minn | Scarlatina. |
| McDannell, O. C | West Point, Iowa | Rheumatism. |
| Morgan, John | Lytle City, Iowa | Life. |
| Olive, Richard | Poinett, Wisconsin | Variola. |
| Orr, R. O. B | Warsaw, Mo | Malarial Fever. |
| Overholt, D. W | Ononwa, Iowa | Semeiology. |
| Perkins, W. F | Monticello, Mo | Digestion. |
| Proctor, J. T | Callio, Mo | Hemostatics. |
| Richards, W. J | Monticello, Ills | Indigestion. |
| Scott, M. B | Etna, Mo | Inflammation. |
| Stevens, J. A | Keokuk, Iowa | The Pulse. |
| Stevenson, Robert | Centreville, Iowa | Typhoid Fever. |
| Stewart, F. C | Iowa City, Iowa | Pneumonia. |
| Stutsman, D. W | Keokuk, Iowa | Erysipelas. |
| Tucker, J. M | Frankfort, Mo | Menstruation. |
| Wall, T. M | Mt. Pleasant Iowa | Chorea. |
| Willhite, W. J | Cambridge, Mo | The Blood in the Heart. |
| Wilson, H. H | Astoria, Ills | The True Physician. |

HONORARY DEGREE.—John J. Oakes, of Mo., and John D. McCleary of Iowa.

All letters in relation to the College, or the admission of patients to the Infirmary, may be addressed to the Dean,

Prof. J. C. HUGHES, M. D., Keokuk, Iowa.

